# Implementing Systematically Collected User Feedback to Increase User Retention in a Mobile App for Self-Management of Low Back Pain: Retrospective Cohort Study

**DOI:** 10.2196/10422

**Published:** 2018-06-06

**Authors:** Innocent Clement, Andreas Lorenz, Bernhard Ulm, Anne Plidschun, Stephan Huber

**Affiliations:** ^1^ Kaia Health Software Munich Germany; ^2^ Department of Orthopaedic Surgery, Physical Medicine and Rehabilitation University Hospital of Munich (LMU), Campus Grosshadern Munich Germany; ^3^ Unabhängige statistische Beratung Bernhard Ulm Munich Germany

**Keywords:** low back pain, app, mHealth, retrospective cohort study, self-management, user feedback, quality management, usability

## Abstract

**Background:**

Promising first results for Kaia, a mobile app digitalizing multidisciplinary rehabilitation for low back pain, were recently published. It remains unclear whether the implementation of user feedback in an updated version of this app leads to desired effects in terms of increased app usage and clinical outcomes.

**Objective:**

The aim is to elucidate the effect on user retention and clinical outcomes of an updated version of the Kaia app where user feedback was included during development.

**Methods:**

User feedback of the initial app versions (0.x) was collected in a quality management system and systematically analyzed to define requirements of a new version. For this study, the anonymized data of Kaia users was analyzed retrospectively and users were grouped depending on the available version at the time of the sign-up (0.x vs 1.x). The effect on the duration of activity of users in the app, the number of completed exercises of each type, and user-reported pain levels were compared.

**Results:**

Overall, data of 1251 users fulfilled the inclusion criteria, of which 196 users signed up using version 0.x and 1055 users signed up with version 1.x. There were significant differences in the demographic parameters for both groups. A log-rank test showed no significant differences for the duration of activity in the app between groups (*P*=.31). Users signing up during availability of the 1.x version completed significantly more exercises of each type in the app (physical exercises: 0.x mean 1.99, SD 1.61 units/week vs 1.x mean 3.15, SD1.72 units/week; *P*<.001; mindfulness exercises: 0.x mean 1.36, SD 1.43 units/week vs 1.x mean 2.42, SD 1.82 units/week; *P*<.001; educational content: 0.x mean 1.51, SD 1.42 units/week vs 1.x mean 2.71, SD 1.89 units/week; *P*<.001). This translated into a stronger decrease in user-reported pain levels in versions 1.x (F1,1233=7.084, *P*=.008).

**Conclusions:**

Despite the limitations of retrospective cohort studies, this study indicates that the implementation of systematically collected user feedback during development of updated versions can contribute to improvements in terms of frequency of use and potentially even clinical endpoints such as pain level. The clinical efficiency of the Kaia app needs to be validated in prospective controlled trials to exclude bias.

## Introduction

Chronic health disorders of the musculoskeletal system such as low back pain are among the conditions with the highest impact on global disability and account for a significant share of direct and indirect costs in health care systems worldwide [1,2]. As many as four out of five people experience low back pain in their lifetime. Furthermore, it is the leading cause of disability worldwide [1] and one of the most frequent reasons for doctor visits [3]. Costs of back pain in most countries are significant economic factors, as the estimate of more than US $90 billion annually spent on low back pain in the United States suggests [4].

The ideal care of low back pain is the subject of ongoing discussion, but therapeutic efforts that emphasize an active role of patients play a key role in the recommendations of international guidelines. Favored strategies for self-management range from exercise therapy and relaxation exercises to constant behavior change via cognitive behavioral therapy [5,6].

However, long-term adherence to any of these strategies is a crucial requirement to achieve enduring clinical benefits for patients. Traditionally, there is a lack of established means to achieve such a goal because long-term behavioral change has received little attention as a means to address chronic diseases such as pain conditions until recently [7]. Recent advances in technological adoption of mobile devices and technological advances in the field have created a promising new strategy aimed at achieving these goals. Mobile health, or mHealth, supports health care methods with the use of apps on mobile phones or tablets. The evidence for its effect is growing in numerous diseases in the form of digital therapeutics [8].

Self-management in the form of digitally supported rehabilitation is a striking candidate for digital interventions supporting patients with musculoskeletal pain [9]. Proof-of-concept studies have consistently shown promising results in terms of clinical endpoints such as pain reduction for patients engaging in the app. At the same time, these studies have shown that long-term engagement is still a challenge [10,11]. A previous retrospective analysis evaluated the effect of the Kaia app, which digitalizes multidisciplinary rehabilitation for self-management of low back pain. It showed that users benefitted from engaging in the app, while the number of users decreased to 32.2% and 17.8% of the original number of users after 8 and 12 weeks, respectively [10].

The involvement of potential users, for example in focus groups, has often been described as a potentially helpful way for early collection of user feedback during app development [12,13]. However, the life cycle of medical apps poses the opportunity to identify and address user feedback in the form of a dynamic and ongoing process even after development and release of first versions [14].

Quality management systems for medical device manufacturers according to standards, such as the European ISO 13485 or US standard 21 CFR part 820, pose a potential framework to systematically collect ongoing user feedback after product release and to document its integration into the development process. The ISO 13485 even requires manufacturers to systematically evaluate feedback with the aim of continuous product improvement [15].

Based on this background, we hypothesized that the implementation of systematically collected user feedback of early versions of the Kaia app for development of updated versions may serve as a potential tool to promote desired outcomes, such as long-term engagement in apps.

In this retrospective study of the Kaia user database, we analyze the effect of the integration of systematically collected user feedback into the development of a new software version on clinical endpoints such as the dropout rate and user-reported pain levels.

## Methods

### Study Design and Users

The study was designed as a retrospective analysis of the user database of Kaia. All users agreed to the collection of data presented in this publication by signing the terms and conditions for use of Kaia. All data used for the study were anonymized for statistical analysis.

The study cohort was recruited via online channels (Facebook, Google Ads, company home page) in Germany, Austria, Switzerland, the United Kingdom, and the United States. The criteria for participation were age older than 18 years, declaration of medical treatment of back pain, no history of indicators for specific causes of back pain (“red flags”), and sufficient level of physical fitness as indicated in the self-test.

The study sample consisted of all users in the user database of the company fulfilling the inclusion criteria listed subsequently.

Users included in the study had to be users of the Pro version of the app because non-Pro users are limited to 1 week of usage only. Users were divided into two groups to reflect whether they signed up to one of the first versions (version 0.x) or version 1.x (starting with 1.4) depending on whether they signed up before or after the release date of version 1.4 (users signing up before April 30, 2017 versus May 1, 2017 or later).

The study was reported to the Institutional Research Board of the Bavarian Regional Medical Council (Bayerische Landesärztekammer) and was waived because of its nonexperimental retrospective and anonymized design.

### Data Collection

#### Data Collection of App Use

All data analyzed in this study were entered by app users as part of their self-test or in-app diaries and stored on company servers in Frankfurt, Germany. Only anonymized data were extracted from the user database via reporting criteria and no personal data were submitted for scientific evaluation.

#### Data Collection of Feedback

All messages from the respective channels that were collected in the quality management system (QMS) were counted and divided into patterns by two different researchers into one metafile on Microsoft Excel.

### Collection of User Feedback in the Quality Management System

Kaia Health Software implemented a QMS for medical device manufacturers that complied with the ISO 13485:2012 standard in September 2016 [15]. In December 2016, the QMS was certified to fulfill the regulatory standards of the ISO 13485 by a notified body (TÜV Süd, Munich, Germany). In line with requirements from the standards of the ISO 13485, a defined analysis for evaluation of user feedback in the form of a standard operating procedure was implemented after initializing the QMS. The channels for user feedback were defined to include in-app coach chat messages, emails to the customer support, comments on app stores, and user surveys.

According to a standard operating procedure, all persons receiving user-related messages continuously monitor all messages to these channels for potential feedback and enter this into the QMS. The original messages containing the feedback are then extracted to metafiles and reviewed during a mandatory feedback meeting held at least twice a year, which systematically collects feedback and transforms it into requirements for the development of further versions of the app. The generation of requirements and evaluation of feedback comments for future versions is continuously monitored by a panel of one managing director of the company, one member of the development team and one member of the quality management team. Metadata of feedback was collected using Microsoft Excel version 12.3.6.

### Description of the Intervention

#### Overall Description of the Intervention

Kaia (Kaia Health Software GmbH, Munich, Germany) is a multiplatform app for iOS, Android, and native Web solutions. Kaia has been on the market since September 2016 and the Kaia app is classified as a medical product class I. It is available via the App Store (iOS), the Google Play Store, or as a native website. Download of the app is free, but to remain active in the app for longer than 7 days, and to unlock the full functionality, users need to upgrade to the Pro version via an in-app purchase.

After downloading the app, there is an explicit sign-up process that consists of questions concerning the user’s previous medical history, pain intensity, and fitness level. Four screens with several items deal with the detection of red-flag conditions that preclude users from exercise in the app as well as medical conditions that pose a contraindication against exercise therapy (see Assessment of Red Flags).

Conditions that require potential urgent medical attention, such as spinal infection or disk herniation associated with neurological deficits, are asked for by the condition names explicitly as well as typical findings in the patient history during the self-test. Patients that show any signs of red-flag conditions in the self-test are only permitted to use the app after they explicitly claim that they have undergone a check-up with a physician and that no contraindication exists that does not permit exercise.

Furthermore, an additional screen asks for the user’s general fitness level to allocate the right exercise difficulty of physiotherapeutic exercises and once more checks for contraindications in terms of insufficient cardiac status. Depending on the results of this initial test, the exercise regimen and content are tailored to the individual user from a pool of exercises based on an algorithm.

Users record their levels of pain on a numeric rating scale (NRS; 0-10 with 10 being the worst imaginable pain) and sleep quality (0-10 with 10 being the best imaginable sleep) at the beginning of each day of therapy in a pain diary as a separate function of the app. Users progress within the app from day to day of practice and the development of user-reported pain and sleep are constantly visible on a screen. There is also a chat function in the app that connects users to a coach (physiotherapist or sport scientist) for motivational and exercise-related questions. A more detailed description of the Kaia app was previously provided [10].

#### Description of the Different Features of Versions

At the end of April 2017, a new version of the Kaia app (version 1.4 and later updates, 1.x) was launched. Requirements for the development of the updated version were generated using user feedback for the previous versions starting the release of Kaia as described previously. Key features of version 1.4 compared to the previous versions (0.x) are listed subsequently.

The updated content features an increased pool of each of the different exercise types (physiotherapy, mindfulness, and education). Furthermore, exercises in each of the categories are customized more clearly to the user’s feedback. Physiotherapeutic exercises are subdivided into 19 different difficulty levels in version 1.4 instead of three different difficulty levels in previous versions. The physiotherapeutic exercises in the Kaia program are exercises based on the concept of lumbar motor control exercise, which has been the subject of many controlled studies [16]. The exact sequence of exercises used in the app, however, has not been scientifically validated before and is based on a consensus of several physiotherapists and sports scientists with experience in pain management programs.

Educational exercises can be chosen from a preselected pool instead of a linear flow. Mindfulness exercises offer a choice of breathing techniques and progressive muscle relaxation after completion of a predefined core set of exercises.

Updates in general app design include customizable reminders, an illustrated sign-up process, a new design, new reminder notifications, and an explanatory introductory day.

### Statistical Analysis

All statistics given for feedback analysis were simple descriptive statistics (absolute numbers or relative numbers) as a fraction of the total of received feedback messages.

A Kaplan-Meier curve with a log-rank test was calculated to estimate the users still active in the app over time. Users still active within 28 days before generation of the dataset were counted as active users and censored.

To analyze the development of pain levels and body, mind, and educational exercises between the two app versions over the weeks, linear mixed effect models were computed.

We opted to use an advanced statistical model, such as the mixed model, in the unbalanced panel dataset because it allows individual-specific inference and is advantageous when dealing with missing values as is the case in this large dataset. Further details on the mixed-model approach have been previously described [17].

Differences in selective time points were also analyzed using *t* tests. For the comparisons of individual values at baseline versus follow-up, we used paired *t* tests. For comparison between groups, nonpaired *t* tests were used. Bonferroni-correction was used adjust for potential multiple test problems. All variables were represented using means and standard deviations. All statistical analyses were done using R version 3.4.3 (November 30, 2017).

## Results

### Analysis of User Feedback and Improvements from Versions 0.x

Overall, 110 unique points of feedback from 41 different users were logged in the QMS during the availability of versions 0.x, 55 (50.0%) of which were submitted using coach chats, 23 (20.9%) using emails, and 32 (29.1%) during user phone calls.

The most frequent parts of the app that were subject of customer feedback were physiotherapeutic exercises with 38 feedback points (34.5%), general app features with 15 feedback points (13.6%), and technical problems during app use with 15 feedback points as well (13.6%).

Of 110 feedback points, 84 (76.4%) were addressed by improvements in versions 1.x in the following release. 23.4% of improvement suggestions from users were not addressed in the development of version 1.4, either due to low priority or because the required effort was deemed too high. Of 16 individual improvements in version 1.4, the improvements addressing most feedback were: better individualization of physiotherapeutic exercises (24 points, 21.8%), new flow of push notifications (10 points, 9.1%), and new educational content and flow of educational section (9 points, 8.2%). Details are shown in Tables 1 and 2.

### Sample Characteristics of Users

Overall, data of 1251 users of the Pro version were available for the study, of which 196 signed up to versions 0.x and 1055 signed up to versions 1.x. The ratio of male to female users differed significantly between the two groups (version 0.x: 58.2%, 114/196 female users; version 1.x: 49.3%, 634/1055 female users, *P*=.03). There was also a significant difference in the mean age of users between the two groups (version 0.x: mean 34.8, SD 11.0 years; version 1.x: mean 45.6, SD 11.6 years, *P*<.001). At baseline, there was no significant difference in pain levels between the two groups (version 0.x: mean 4.41, SD 1.57 NRS; version 1.x: mean 4.19, SD 1.55 NRS, *P*=.08).

### Dropout of Users Over Time

**Figure 1:**
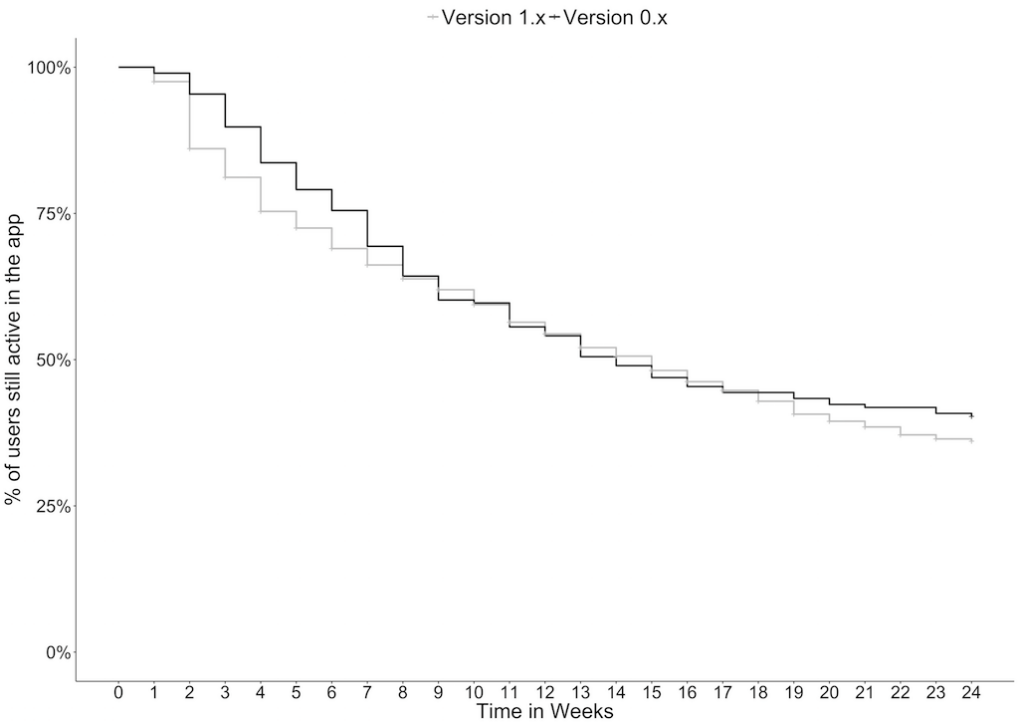
Kaplan-Meier plot of dropout of users in percentage over time in weeks.

We assessed whether users were still active in the app by signing on and finishing at least one exercise type each week following sign-up. The results for both groups are shown in a Kaplan-Meier-plot (Figure 1; Table 3). A log-rank test revealed no significant difference in dropout for users of the two groups (*P*=.31), indicating a comparable rate of dropouts over time for both groups, whereas many users in the version 1.x cohort had to be censored because they were still counted as active users by definition.

### Completion of Exercises by Type

To assess whether there was a difference in the rates of exercise completion, we analyzed the rate of weekly units per user and week for each of the different types of content (physical and mindfulness exercises, educational units).

**Table 1:** Domains of app affected by feedback.

Domain	Messages, n (%)
Physiotherapeutic exercises	38 (34.5)
Mindfulness exercises	12 (10.9)
Educational content	9 (8.2)
Notifications	10 (9.1)
Pain diary and self-test	4 (3.6)
Chat	1 (0.9)
Design features	2 (1.8)
General app features	15 (13.6)
Technical problems	15 (13.6)

**Table 2:** Improvements in version 1.x with number of feedback issues fixed by individual improvements.

Improvement	Feedback messages fixed by improvement, n (%)
Not implemented	26 (23.6)
New educational content	9 (8.2)
New in-app flow to access content	4 (3.6)
Audio files stored locally on client	2 (1.8)
New content for physiotherapy	3 (2.7)
New flow for push notifications	10 (9.1)
Better adaptation of physiotherapeutic exercises to individual user	24 (21.8)
New concept for daily renewal of content	4 (3.6)
Increase stability on Android devices with testing on several devices	1 (0.9)
New audio content for physio exercises	5 (4.5)
Show more clearly how app adapts to user feedback	2 (1.8)
Redesign self-test questions	1 (0.9)
Real-time synchronization of data	6 (5.5)
Record new and extended content for mindfulness exercises	6 (5.5)
Fix password reset option	1 (0.9)
Exchange library for video playback	5 (4.5)

**Table 3:** Weekly active users, dropouts, and censored users for both groups.

Week	Versions 0.x			Versions 1.x					
	Active users, n	Dropouts, n	Users still active, %		Active users, n	Dropouts, n	Censored, n	Cumulative censored, n	Users still active, %
1	196	2	99.0%		1055	26	8	8	97.5%
2	194	7	95.4%		1021	120	58	66	86.1%
3	187	11	89.8%		843	48	42	108	81.2%
4	176	12	83.7%		753	54	37	145	75.4%
5	164	9	79.1%		662	25	38	183	72.5%
6	155	7	75.5%		599	29	29	212	69.0%
7	148	12	69.4%		541	22	17	229	66.2%
8	136	10	64.3%		502	18	38	267	63.8%
9	126	8	60.2%		446	13	23	290	62.0%
10	118	1	59.7%		410	17	35	325	59.4%
11	117	8	55.6%		358	18	28	353	56.4%
12	109	3	54.1%		312	11	23	376	54.4%
13	106	7	50.5%		278	12	16	392	52.1%
14	99	3	49.0%		250	7	16	408	50.6%
15	96	4	46.9%		227	11	15	423	48.2%
16	92	3	45.4%		201	8	7	430	46.2%
17	89	2	44.4%		186	6	11	441	44.7%
18	89	0	44.4%		169	7	7	448	42.9%
19	87	2	43.4%		155	8	12	460	40.7%
20	85	2	42.3%		135	4	10	470	39.5%
21	83	1	41.8%		121	3	4	474	38.5%
22	83	0	41.8%		114	4	3	477	37.1%
23	82	2	40.8%		107	2	8	485	36.4%
24	80	1	40.3%		97	1	96	581	36.1%

**Figure 2:**
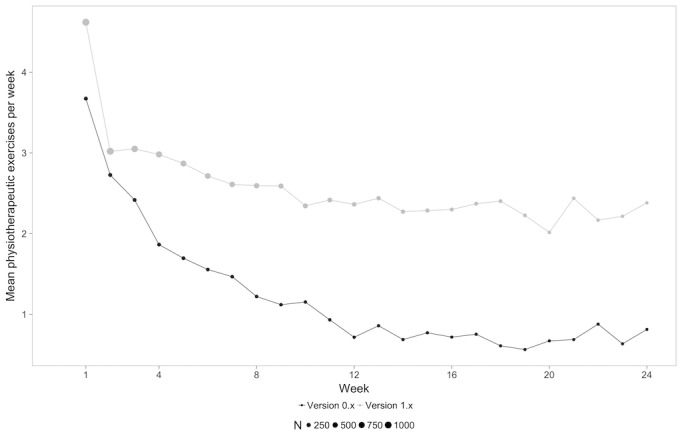
Mean physiotherapeutic exercises per week for each content type over time for both groups.

**Figure 3:**
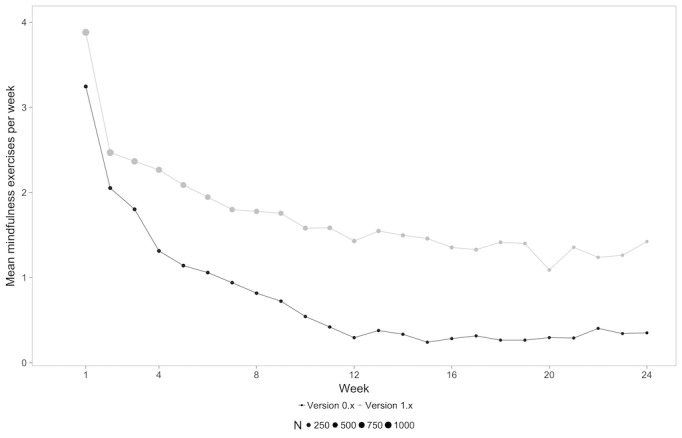
Mean mindfulness exercises per week for each content type over time for both groups.

**Figure 4:**
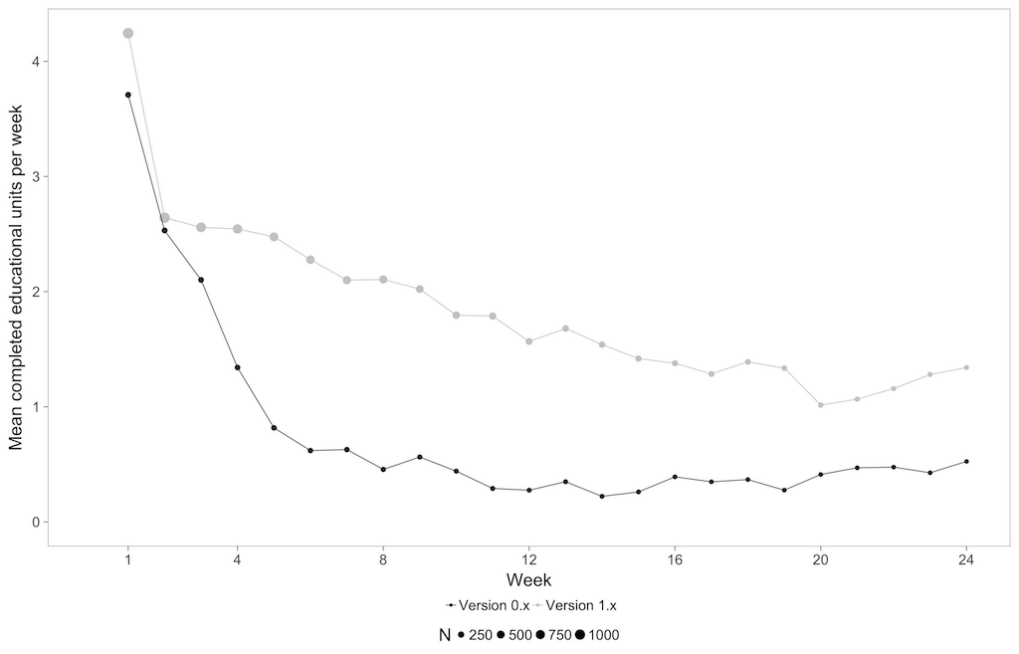
Mean educational exercises per week for each content type over time for both groups.

A mixed-model analysis revealed a significant difference between groups for all types of content (physical exercises: *F*_1,1249_=52.303, *P*<.001; mindfulness exercises: *F*_1,1249_=28.62, *P*<.001; educational units: *F*_1,1249_=42.891, *P*<.001), indicating that users in the 1.x group completed more of each unit in each category. The amount of completed units for each type of content for each week is shown in Figures 2-4.

This finding was also confirmed when comparing mean values of each of the types of content averaged over the 24 weeks with a paired *t* test (physical exercises: version 0.x mean 1.99, SD 1.61 units/week vs version 1.x mean 3.15, SD 1.72 units/week, *P*<.001; mindfulness exercises: version 0.x mean 1.36, SD 1.43 units/week vs version 1.x mean 2.42, SD 1.82 units/week, *P*<.001; educational content: version 0.x mean 1.51, SD 1.42 units/week vs version 1.x mean 2.71, SD 1.89 units/week, *P*<.001).

### In-App Reported Pain Levels

**Figure 5:**
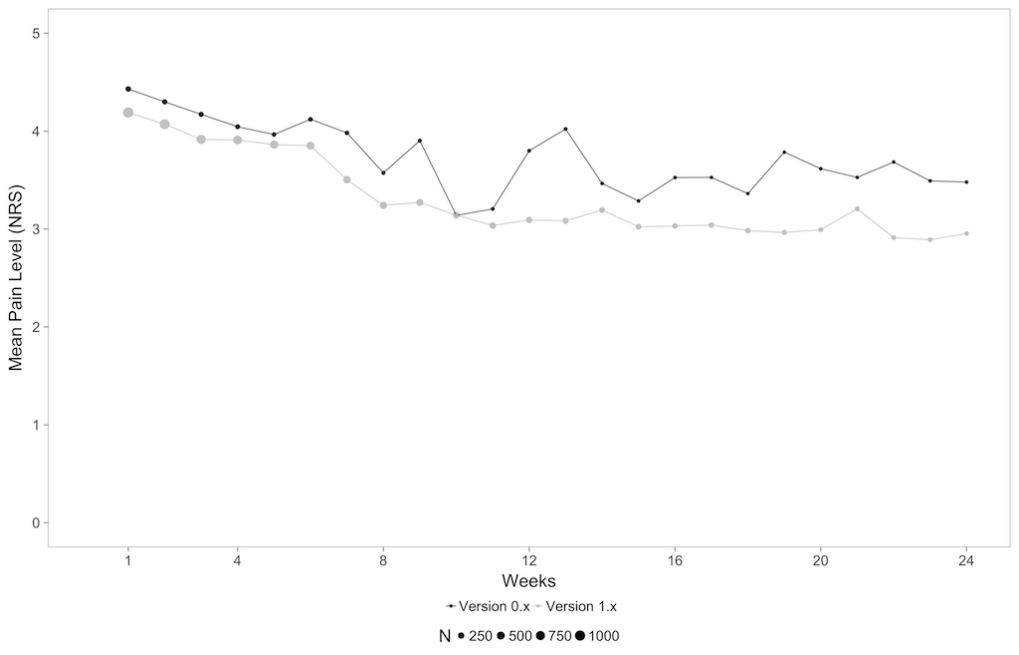
In-app reported pain levels of users on the NRS for both versions over time (weeks).

To see, whether the increase in completed units translated into a significant benefit in terms of the clinical endpoint of pain as reported on the NRS, we assessed pain levels over time for both groups. A mixed-model analysis showed a significant difference between groups that was indicative of an increased reduction in terms of user-reported pain levels in the 1.x group (*F*_1,1233_=7.084, *P*=.008). There was a decrease in pain levels from baseline to 24 weeks in both groups (mean 4.4, SD 1.5 at baseline vs mean 3.48, SD 2.1 at week 24 for version 0.x; mean 4.2, SD 1.6 at baseline vs mean 3.0, SD 2.1 at week 24 for version 1.x; *P*=.29). The course of user-reported pain levels over time is depicted in Figure 5 and Table 4.

**Table 4:** Mean weekly in-app reported pain levels at baseline and at 12 and 24 weeks.

Week	Version 0.x (n=158), mean (SD)	Version 1.x (n=998), mean (SD)	*P* value^a^
1 (baseline)	4.43 (1.50)	4.19 (1.55)	.06
12	3.80 (2.17)	3.09 (1.78)	.07
24	3.48 (2.09)	2.95 (2.17)	.29

^a^*P* values are for nonpaired *t* test between groups.

## Discussion

### Findings of the Study

The development of a new version of an app integrating user feedback for self-management of low back pain can increase app use significantly and thus increase clinical benefit in terms of self-reported pain levels. The analysis of user data in this study reveals that users signing up for the updated version of the Kaia app did not remain active in the app for longer periods of time, but engaged in the individual exercises more often than users of the previous version. Of note, this translated into an increased benefit concerning pain levels as a clinical endpoint. Therefore, within the limitations of this retrospective report of user data, we propose that implementing systematically collected user feedback can serve as an effective measure to actively engage users in an app for self-management and even improve the clinical endpoint of user-reported pain.

The dropout rate in this study was significantly lower than that reported in a previous study on the Kaia app [10]. However, even though dropout rates improved in comparison to previous reports, they remain an incentive for further improvement of the Kaia app. In addition to remaining feedback from users still not accounted for in version 1.x, there are other factors potentially explaining the dropouts. On the one hand, the app features no long-term rewards or group interaction that may further increase retention. On the other hand, a prospective study design with inclusion in centers and defined follow-up visits may also contribute to higher retention in studies of other interventions, but are not present in the real-world data of this study.

This most likely reflects the fact that many users, who were listed as inactive in the previous study, became active again afterwards and the user retention improved with the new version of the app. Of note, the different duration of the observational period for both groups also introduces a potential bias in this analysis because users in the version 0.x group progressed over a longer period of time in the app. Nevertheless, the short-term rate of dropouts is comparable to observed rates for other interventions to treat musculoskeletal pain conditions with apps in a recent study [18]. The midterm retention at 24-weeks in this study shows that a significant proportion of users still engage in the app and that completion of body exercises per week only slightly decreases in app users following the first few weeks.

The pattern of users remaining engaged in the app also decreasing their pain levels on the NRS and retaining that benefit over time has been described before in a number of noninterventional studies evaluating the effect of digital interventions for rehabilitation of pain conditions [10,11,18]. The follow-up of this study is comparatively long and indicates that the self-management strategies conveyed by the Kaia intervention may indeed induce stable pain reduction well above the minimal clinically important difference for the NRS for chronic orthopedic pain conditions [19,20] with an absolute improvement of more than one point on the NRS and almost 30% relative improvement, which almost reaches the threshold for “much improved” musculoskeletal pain conditions [20].

However, given the limitations of this study, this finding will need to be reproduced by prospective studies. The authors would like to point out that, although current meta-analysis articles have found little evidence for a clinical effect of digital interventions in low back pain, the interventions in these reports have mostly consisted of cognitive behavioral therapy that was delivered via Web interfaces. None of the interventions in those studies focused on a multidisciplinary rehabilitative approach delivered via mHealth; therefore, they did not make use of the full potential of digital interventions in terms of content or design [21,22].

Digitalized self-management strategies offer a promising novel strategy for long-term patient engagement in chronic diseases. Retention and continuous behavioral change are crucial for this goal [8]. Some features, such as the inclusion of health care professionals, individualization of the app, and user-friendly design, have been found to increase the effectiveness of digital interventions for this goal in a recent review [23].

Integrating users in the design of apps and the continuous improvement process have been recognized as a crucial factor for app adoption. Various techniques, such as participatory design, have been developed for early user integration in development [24].

Many reports have previously described the collection of the feedback of prospective users in early app development or during focus groups [12,13,25]. In case of multidisciplinary concepts for disease management of musculoskeletal pain conditions, potential users interviewed in focus groups have indicated motivational traits, an introductory feature, and individualization to be important features [13]. Notably, all these topics were also mentioned as potential improvements by users of Kaia.

Compared to focus groups and other structured interviews, the collection of real-world feedback of users, as used in this study, offers many advantages as an alternative approach for participatory design. Most importantly, this approach makes use of the rapid development cycles of health apps in which potential improvements can be integrated quickly. Furthermore, potential users have often already spent a significant amount of time with the software at home, where the software is intended for use. Therefore, feedback collected from real-world settings is likely to better reflect the problems encountered by users in their everyday patient journeys using digital interventions.

Another study has evaluated user preferences for desirable features of health apps and found structure, ease of use, personalized features, and accessibility to be the most important features for users [26]. Indeed, user feedback concerning the Kaia app dealt with many of these issues. Redefinition of structure, personalization of content in each of the categories, and simplification of app use were among the most frequent user requests, and all were addressed with new content or features in the novel 1.4 version.

### Limitations

Limitations of this study arise for the largest part from the design as a retrospective cohort study. This design makes the study vulnerable for potential bias in the form of selection bias. There is also a significantly different demographic composition of the two cohorts compared in the study. Also, by definition, it is difficult to compare different types of cohorts with one another when no randomization has taken place. The methodology to compare cohorts with different observational periods makes the Kaplan-Meier plots less representative and introduces a potential bias that is likely to overestimate the dropout rate in the group with the shorter observational period. Because this analysis contains only very few users older than 60 years of age, this study cannot draw any conclusions about the efficacy and retention of the Kaia app in a population older than 60 years. This is a limitation of this study that should be addressed by prospective studies in selected populations of users older than 60 years.

### Conclusions

This study indicates that, given the limitations of retrospective cohort studies, the implementation of systematically collected user feedback during development of updated versions can contribute to improvements in terms of use frequency and potentially even clinical endpoints such as pain level. The clinical efficacy of the Kaia app needs to be validated in prospective controlled trials to exclude bias.

## Abbreviations

NRS: numeric rating scale
QMS: quality management system
